# NFκB signalling in colorectal cancer: Examining the central dogma of IKKα and IKKβ signalling

**DOI:** 10.1016/j.heliyon.2024.e32904

**Published:** 2024-06-12

**Authors:** Molly McKenzie, Guang-Yu Lian, Kathryn A.F. Pennel, Jean A. Quinn, Nigel B. Jamieson, Joanne Edwards

**Affiliations:** School of Cancer Sciences, Wolfson Wohl Cancer Research Centre, University of Glasgow, Glasgow, G61 1BD, UK

**Keywords:** Colorectal cancer, Cell signalling, NFκB, IKKα, IKKβ, NIK, Inflammation, Prognosis, Biomarkers, Tumour microenvironment

## Abstract

The NFκB pathway, known as the central regulator of inflammation, has a well-established role in colorectal cancer (CRC) initiation, progression, and therapy resistance. Due to the pathway's overarching roles in CRC, there have been efforts to characterise NFκB family members and target the pathway for therapeutic intervention. Initial research illustrated that the canonical NFκB pathway, driven by central kinase IKKβ, was a promising target for drug intervention. However, dose limiting toxicities and specificity concerns have resulted in failure of IKKβ inhibitors in clinical trials. The field has turned to look at targeting the less dominant kinase, IKKα, which along with NFκB inducing kinase (NIK), drives the lesser researched non-canonical NFκB pathway. However prognostic studies of the non-canonical pathway have produced conflicting results. There is emerging evidence that IKKα is involved in other signalling pathways, which lie outside of canonical and non-canonical NFκB signalling. Evidence suggests that some of these alternative pathways involve a truncated form of IKKα, and this may drive poor cancer-specific survival in CRC. This review aims to explore the multiple components of NFκB signalling, highlighting that NIK may be the central kinase for non-canonical NFκB signalling, and that IKKα is involved in novel pathways which promote CRC.

## Highlights

• The NFκB pathway has a well-established role in driving tumour initiation and progression.
• Recent work has suggested NIK is the central kinase for non-canonical signalling and IKKα may have roles in alternative pathways.
• In colorectal tumours this has been shown to be through p45-IKK driven signalling.
• Research is now focused on targeting the non-canonical pathway, through NIK, IKKα or alternative pathway inhibition.

## Graphical abstract

**Figure:**
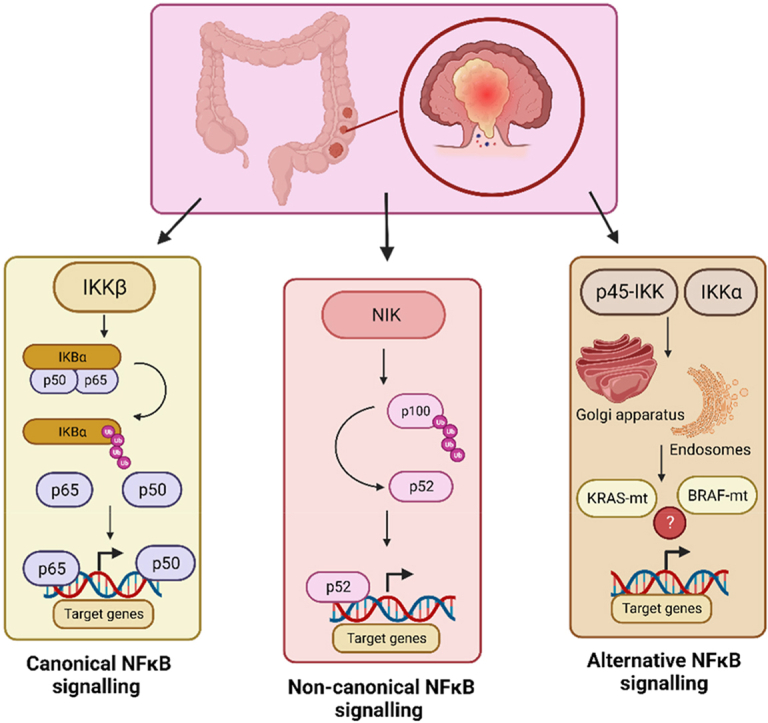

## 1 Introduction

As the second most lethal and the third most prevalent cancer [1], colorectal cancer (CRC) poses a significant healthcare issue. Due to advances in screening and therapies, CRC death rates have decreased by ∼50 % over the past 50 years. However, the 5- year survival rate for CRC is only ∼65 %, which falls to 12 % for metastatic disease [2] and incidence of CRC is increasing in high-income countries and in adults under 50 years of age [3,4].

Due to the heterogeneous nature of CRC, its treatment is often multimodal, accounting for location, stage, metastasis, mutational status and biomarkers present [5], as well as the established factor that colon and rectal cancer should be considered as different diseases [6,7]. Targeted therapies such as monoclonal antibodies cetuximab and bevacizumab, or the immunotherapy pembrolizumab, have advanced the treatment of both colon and rectal cancer, producing longer progression-free survival and fewer side effects than chemotherapy [[8], [9], [10]]. However key issues remain in the treatment of both colon and rectal cancer; therapies are only effective in specific subgroups of patients, with many experiencing intolerable side effects, and therapy resistance [11,12]. Furthermore, key differences between disease sites can drive different prognoses and effectiveness of treatment; with rectal cancers more likely to benefit from neoadjuvant therapy [13], and right-sided colon cancer, associated with an immune cell rich environment, demonstrating the poorest patient outcome in CRC [14]. There is a clear need for novel therapeutic agents to tackle unmet needs of colon and rectal cancer treatment.

In 2011, Hanahan and Weinberg introduced tumour-promoting inflammation as an enabling characteristic in the context of the landmark paper, Hallmarks of Cancer: The Next Generation [15]. While local inflammation, such as a high influx of immune cells, is associated with good patient outcome [16], systemic inflammation has cemented its role as a driver of tumour development and a viable target for therapeutics [17]. Both colon and rectal cancer have a well-established association with systemic inflammation, with evidence suggesting three key areas of CRC inflammation; chronic inflammation promoting tumorigenesis; tumour-produced inflammation driving cancer hallmarks, and paradoxically; inflammation produced in response to therapy [[18], [19], [20]].

### 1.1 NFκB

**Fig. 1:**
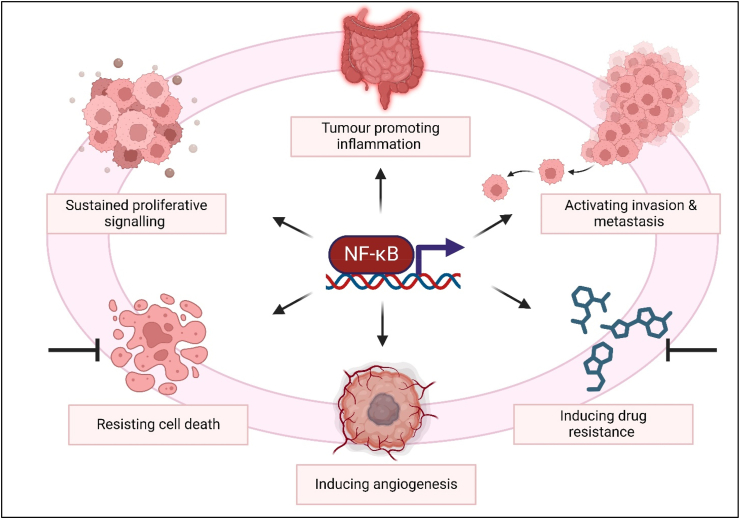
**NFκB in Colorectal Cancer**; figure illustrates the multiple hallmarks of cancer which are associated with NFκB signalling. Although usually associated with inflammation, it is clear that NFκB signalling has reaches across the cancer hallmarks, making it an attractive target for therapeutic intervention. Created in Biorender. com.

The nuclear factor kappa-light-chain enhancer of activated B cells (NFκB) transcription factor is a central regulator of the inflammatory response [21], and is key for CRC inflammation. NFκB activation promotes not only inflammation [22] but tumour proliferation [23], cell death evasion [24], angiogenesis, metastasis [25] and inflammation-based mechanisms of drug resistance [26,27], as illustrated in Fig. 1. Targeting the NFκB pathway has become a focus of the field, especially investigating members of the inhibitor of kappa B kinase (IKK) family, IKKα and IKKβ, which function as inhibitors of NFκB inhibitory proteins. IKKα joins with fellow kinase IKKβ, and substrate specificity factor, NFκB essential modulator (NEMO), to form the IKK complex, regulating NFκB activation, through canonical and noncanonical signalling pathways [28,29].

Traditionally IKKβ has been identified as the more dominant IKK, especially in terms of canonical signalling, however, despite significant investigation, IKKβ inhibitors are yet to gain clinical approval due to resulting toxicities, comprehensively outlined by Ramadass et al. [Ramdass et al., 2020].

Conversely, there is a growing body of evidence demonstrating that IKKα is as critical in NFκB signalling [30,31]. Targeting this kinase may be able to accomplish the desired inhibition of NFκB signalling, without systemic toxicities associated with IKKβ inhibition [32]. However, there is a need to understand the role of IKKα within the tumour and the tumour microenvironment (TME), as well as robust testing of preclinical IKKα inhibitors, and identification of which CRC patient populations could benefit from targeted inhibition before the clinical translation of IKKα inhibitors.

This review aims to provide an overview of the evolving discussion around IKK signalling in CRC; highlighting how the presence of alternative signalling pathways outside of the canonical and non-canonical central dogma may provide novel avenues for therapeutic intervention for CRC patients.

## 2 NFκB family proteins and their regulation

**Fig. 2:**
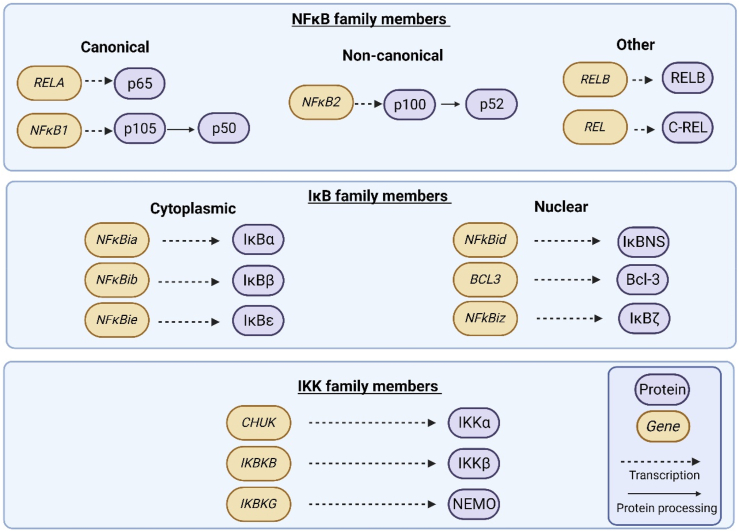
**Members of the NFκB, IκB and IKK families**, illustrating the gene transcription, and subsequent protein processing, and resulting active proteins. The 5 proteins of the NFκB family are closely regulated by that of the IκB family. This inhibitory regulation can be removed by IKK family members, namely IKKα and IKKβ. **Abbreviations:** B-Cell Lymphoma-3 (Bcl-3), NFκB inhibitor alpha (IκBα), NFκB inhibitor beta (IκBβ), NFκB inhibitor delta (IκBNS), NFκB inhibitor epsilon (NFκϵ), NFκB inhibitor zeta (NFκζ), Inhibitor of nuclear factor kappa-B-kinase subunit alpha (IKKα), Inhibitor of nuclear factor kappa-B-kinase subunit alpha (IKKβ), Inhibitor of nuclear factor kappa B kinase essential modulator (NEMO). Created in Biorender. com.

Signalling of the IKK proteins is closely intertwined with that of NFκB- a family of highly regulated transcription factors, responsible for the translation of pro-tumour genes- and with its regulators, the inhibitor of NFκB (IkB) family of proteins, as illustrated in Fig. 2. The interplay within these three families of proteins shapes the translational landscape of over 150 genes [33,34].

### 2.1 NFκB proteins

Discovered in 1986 as a B cell-specific transcription factor [35], the NFκB family are an integral group of inducible transcription factors, expressed across nearly all cell types as the pleiotropic end of multiple signalling pathways, often co-opted in cancer development [36]. The NFκB family consists of 5 members, which dimerise in protein form to propagate distinct signalling pathways: p65, p105/50, p100/p52, RELB and C-REL [37]. Traditionally p65 and p105/50 have been associated with canonical signalling, and p100/p52 with non-canonical.

Referred to as the NFκB subfamily within the NFκB family of proteins, p105 and p100 are both precursor proteins which are cleaved into active forms. Encoded by the gene nuclear factor kappa B subunit 1, the precursor protein p105 undergoes cleavage by 20 S, the catalytic core of the 26 S proteasome, resulting in p50 [38]. This occurs in a ubiquitin-independent manner, where the c-terminus of p105 is degraded, processing p105 into p50, a key component for forming NFκB hetero/homodimers. This process is active within most cells and occurs at a co-translational level, highlighting the constitutive nature of NFκB canonical signalling [39].

The second of the NFκB subunit genes (*NFκB 2*), encodes a further set of precursors and cleaved proteins, where p100 is co-translationally processed into p52, which can act as both a transcriptional activator and repressor dependent on the dimer formed [40]. Due to the majority of NFκB processing studies being carried out on p105/p50, less is known about p100/52 processing. However, it has been suggested that p100 processing occurs at a less frequent rate than p105, with less p52 being produced in cells than p50 [41].

Within the Rel subfamily of the NFκB family, p65 (also known as RelA), is encoded by the *RELA* gene. This contains an N-terminal REL-homology domain (RHD), present in all NFκB family proteins, and C-terminal transactivation domain, involved in DNA binding, dimerization, and translocation into the nucleus, as well as interactions with transcription co-activators respectively [42]. This protein dimerises with the processed protein p50, to form the most common NFκB heterodimer.

The other Rel members include RelB and C-REL. The *RELB* gene encodes the transcription factor RelB, an unstable protein which forms dimers with NFκB subfamily proteins to avoid degradation. Within these dimers, RelB can exert both an inhibitory or promoting influence for NFκB elicited gene expression, with evidence highlighting RelB's wide-reaching functions and roles [43]. RelB is associated with non-canonical NFκB activity, which is explored in later sections of the review. Finally, C-REL, which in contrast to its widespread family members, is exclusively highly expressed in B and T cells, in which it is critical for lymphoid maturation [44].

Interestingly, there are varying regulatory effects between the NFκB proteins themselves. Within both p105 and p100 processing, the degraded c-terminus contains ankyrin repeat domains. These domains can dimerise with other NFκB family members and inhibit their activity, as well as prevent translocation into the nucleus [45]. This has been elegantly illustrated in terms of p105 inhibitory signalling, where p105 was shown to retain C-REL and p65 within the cytoplasm [46]. Additionally, this inhibitory effect has also been investigated in terms of p100. One such study utilised mouse embryonic fibroblasts, knocking out; IkBα, IkBβ and IkBɛ, illustrating that in the absence of these key regulatory proteins, p65 did not completely move into the nucleus as expected. They demonstrated that p100 could bind to p65:p50 dimers, retaining them in the cytoplasm in response to IKKα activating stimuli. This inhibitory role of p100 illustrated its ability to act as a regulator across both the canonical and non-canonical pathway, highlighting that these pathways should not be considered as separate dogmas, but able to cross talk dynamically dependant on stimuli [47].

### 2.2 IkB proteins

Through the multiple dimer pairings and redundant nature of NFκB signalling, which feeds into the last steps of multiple signalling cascades, NFκB signalling requires strict regulation. This is carried out by the IkB protein family, consisting of IkBα, IkBβ and IkBɛ within the cytoplasm, and IκBζ, IκBNS, Bcl-3 within the nucleus [48,49]. IkB proteins contain ANK repeat domains, which form cylindrical structures to specifically bind to RHDs present on NFκB proteins, inhibiting their entry into the nucleus [42,50].

A positive feedback loop maintains this level of regulation, as transcription targets of active NFκB include the genes *NFκBia* and *NfκBie*, encoding IkBα and IkBɛ respectively. Once transcribed, these IkB proteins have overlapping yet distinct methods of regulating NFκB. As outlined by Hoffmann's computational model, fast transcription and therefore fast inhibitory action is mediated by IkBα, whereas the more delayed, slower transcription and resulting NFκB inhibition is mediated by IkBβ and IkBɛ [51].

In contrast, IkBβ has also been shown to undo the inhibitory effect of IkBα through binding p65 and C-REL, allowing for these transcription factors to become active. This effect can be inhibited through IkBβ phosphorylation [52]. This dynamic relationship within the IkB family of proteins for NFκB regulation highlights the varied and important nature of NFκB signalling.

However, IκBα has also been shown to have roles outside of NFκB signalling. Within keratinocytes, a form of IκBα which has undergone SUMOylation has been shown to directly interact with the chromatin modulator polycomb repressive complex 2 (PRC2), repressing targets such as HOX genes [53]. This can be relieved via stimulation by cytokines such as TNFα, leading to a high level of HOX gene activation, which is hypothesised to promote tumour development and oncogenesis [54]. This NFκB independent role of IκBα once again highlights the need to consider the roles of NFκB proteins outside of canonical and non-canonical signalling.

### 2.3 IKK proteins

Finally, regulating the IkB family of proteins, are the IKKs. Characterised in 1997, this family of proteins consists of IKKα, IKKβ and NFκB essential modulator (NEMO), also referred to as IKK1, IKK2 and IKKγ respectively [55,56].

Encoded by the component of inhibitor of nuclear factor kappa B kinase complex (*CHUK*) gene, IKKα functions as an inhibitor of the NFκB inhibitors. This consists of a kinase activation loop at Serine 176 and 180, a regulatory helix loop helix domain, a leucine zipper which allows for dimerization with other IKK subunits, and a carboxy-terminal NEMO binding domain [57,58].

The other kinase subunit, IKKβ, is encoded by the inhibitor of the nuclear factor kappa B kinase subunit beta (*IKBKB*) gene. Sharing over 50 % of gene sequences, IKKβ has the same structure as IKKα, with its activation loop found at serine 177 and 188. Crystal structure analysis has shown IKKβ also contains a ubiquitin-like domain, which is critical for the kinase's catalytic activity and substrate interaction [59].

NEMO, a regulatory protein required for IKK activation [60], is encoded by the inhibitor of nuclear factor kappa B kinase regulatory subunit gamma (*IKBKG*) gene. Distinct from the two kinases, NEMO is structurally different, containing a leucine zipper, zinc finger domain and two coiled-coil domains which facilitates binding with the kinases [61].

The activation of IKKα and IKKβ and their interactions with NEMO, facilitate the degradation of IκB proteins, allowing for NFκB proteins members, such as p50 and p65, to translocate into the nucleus and initiate the transcription of target genes. The interplay between these three families- NFκB, IκB and IKK- has been well studied and from this two distinct methods of NFκB activation have emerged; the canonical and non-canonical pathways.

## 3 The central dogma of NFκB signalling canonical and non-canonical signalling

Since the discovery of NFκB and its associated proteins, studies have elucidated two main pathways of pathway activation, each driven by one of the IKKs; the canonical signalling pathway, modulated by IKKβ and the non-canonical pathway, driven by IKKα. These pathways have formed the central dogma of NFκB signalling, guiding the development of drugs targeting NFκB activation [62].

### 3.1 Canonical NFκB signalling

**Fig. 3:**
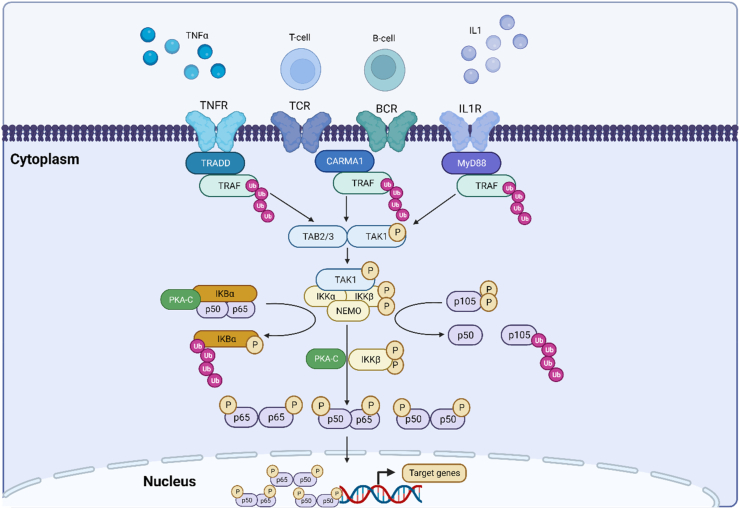
**The NFκB canonical signalling pathway:** the canonical signalling pathway can be activated by numerous signalling ligands and immune cells, to produce a rapid and transient inflammatory response. Supported by adaptor and scaffolding proteins (TRADD, CARMA1, MyD88), TRAF1 is activated, resulting in its self ubiquitination, and subsequent activation ofTAK1. TAK1 forms a complex with TAB2/3, allowing for the phosphorylation of IKKβ. TAK1 forms a complex with the IKKs (IKKα, IKKβ and NEMO). The now activated IKKβ phosphorylates IkBα, leading to its ubiquitination and proteasome-based degradation. IKKβ also phosphorylates p105 at Ser 927 and Ser932, targeting it for ubiquitination and partial degradation to generate p50. Free p50 and p65 form homo/heterodimers, which enter the nucleus-this process is enhanced by PKA-C and IKKβ driven phosphorylation of p50 and p65. Once in the nucleus, p50 and p65 bind to DNA response elements, facilitating the transcription of pro-inflammatory genes. **Abbreviations:** B-cell receptor (BCR), Caspase recruitment domain containing protein 1 (CARMA1), Inhibitor of nuclear factor kappa B kinase essential modulator (NEMO), Inhibitor of nuclear factor kappa-B-kinase subunit alpha (IKKα), Inhibitor of nuclear factor kappa-B-kinase subunit alpha (IKKβ), Interleukin-1 (IL1), Interleukin-1 receptor (IL1R), Myeloid differentiation primary response 88 (MyD88), NFκB inhibitor alpha (IκBα), Protein kinase A catalytic subunit (PKA-C), T-cell receptor (TCR), TGF-Beta activated kinase binding protein 2/3 (TAB2/3), TGF-Beta-Activated Kinase 1 (TAK1), Tumour necrosis factor alpha (TNFα), Tumour necrosis factor receptor (TNFR), Tumour necrosis factor receptor associated factor (TRAF), Tumour necrosis factor receptor type-1 associated death domain (TRADD). Created in Biorender. com.

Classically considered the dominant pathway of NFκB activation, the canonical signalling pathway can be activated by a diverse range of stimuli, leading to fast and transient activation of the pathway, as shown in Fig. 3. These include ligands stimulating the tumour necrosis factor receptor (TNFR), T-cell receptors, B-cell receptors, pattern-recognition receptors, and pro-inflammatory cytokine receptors, such as IL-1R [63]. This variation in activating stimuli allows for the canonical NFκB pathway to respond to signals from both the adaptive and innate immune systems, described in detail by Yu et al. [64].

Upon stimulation of a participating receptor, the TGFβ activated kinase 1 (TAK1) is activated, usually via activation and subsequent self-ubiquitination of a TNFR-associated factor (TRAF) family protein. This is facilitated by an array of signalling proteins such as TRADD, CARMA1 and MyD88, depending on the receptor stimulated [65].

Once activated, TAK1 forms a complex with the IKK proteins (consisting of IKKα, IKKβ and NEMO) where TAK1 phosphorylates IKKβ at Ser171, inducing IKKβ autophosphorylation at Ser181 [66]. The formation of this complex is facilitated by NEMO, often modulated by post-translational modifications, such as the binding of Met1-linked ubiquitin oligomers to NEMO [67], or lysine-63-linked ubiquitination under Bcl10 driven signalling [68]. The formation of these linear ubiquitin chains is regulated by the linear ubiquitin chain assembly complex, consisting of a catalytic subunit known as HOIP (Hoil-1 interacting protein) [69]. There are also examples of NEMO activating IKK proteins within the nucleus, such as under conditions of genotoxic stress; where unbound NEMO undergoes a SUMO-1 modification and is translocated into the nucleus. Once within the nucleus, NEMO is ubiquitinated, which interestingly leads to IKK activation within the cytoplasm [70].

The now activated IKKβ phosphorylates IkBα at Ser32 and Ser36, leading to its ubiquitination and proteasome-based degradation. This removes the inhibition on NFκB proteins p50 and p65, as they have been held in an inactive form in the cytoplasm by IkBα [71].

IKKβ also phosphorylates p105 at Ser927 and Ser932, leading to its ubiquitination and partial degradation to generate p50 [72]. This is thought to contribute to cytoplasmic p50 levels, as this protein is also produced at a co-translational level. However, there have been conflicting reports regarding IKKβ driven processing of p105 resulting in active p50 [73,74].

In the majority of cell types, free p50 proteins form heterodimers with p65, while p50/p50 and p65/p65 homodimers are less common. The specific composition of these dimers can have functional impact on the transcription of NFκB target genes as only p65, RelB and C-REL contain a transcriptional activation domain (TAD), essential for promoting transcription. Those NFκB subunits lacking TAD can repress transcription [75]; for example, p50/p50 homodimers have been shown to suppress gene expression through forming complexes with the histone deacetylase HDAC-1 [76,77].

p65 can be phosphorylated at multiple serine and tyrosine residues, resulting in conformational changes, enhancing nuclear import, increasing promoter binding and producing greater transcriptional activity [78]. Currently eleven phosphorylation sites on p65 have been identified, the two most widely studied being Ser276 and Ser536; targeted by protein kinase A and IKKβ respectively. Work by Zhong et al. illustrated that protein kinase A's catalytic subunit (PKA-C) is also kept in an inactive state by IκBα. Upon IκBα degradation, the now free PKA-C phosphorylates p65 at serine 276, resulting in increased transcriptional activity [79,80]. Phosphorylation of p65 at S536 can be attributed to multiple kinases, such as IKKα and IKKβ, with the induction of this phosphorylation has been shown to be tissue-specific, as extensively outlined by Christian et al. [72]. This phosphorylation has been demonstrated to increase transactivation of p65, by allowing for p65 acetylation by histone acetyltransferase p300 [81,82].

Additionally, phosphorylation of p50 is important within the canonical NFκB pathway. Lesser studied than its p65 counterpart, p50 has five known phosphorylation sites: Ser337, Ser328, Ser242, Ser20 and Ser80 [83]. Phosphorylation by PKA at the Ser337 site has been shown to be important for DNA binding and supressing transcription, while phosphorylation at Ser242 has been shown to reduce DNA binding [84]. Additionally, S328 phosphorylation sites regulate p50's role in the DNA damage response, and S20 regulates VCAM-1 expression in response to TNFα [85]. S80, the most recently discovered phosphorylation site, is a substrate of IKKβ kinase activity. Upon TNFα stimulation, the phosphorylation of IKKβ at S80 regulates transcription of a specific set of NFκB target genes [83].

These post-translational modifications add a further layer of regulation to the NFκB pathway, highlighting that the pathway can result in many different responses, dictated by the cell type and stimuli present, exerting versatile functions across disease and tissue type.

Upon phosphorylation the p65/p50 complex is then translocated into the nucleus via importin α3/4, which recognise and bind to nuclear localisation sequences (NLS) present on both p50 and p65 [86]. Within the nucleus, these NFκB proteins bind to DNA response elements to facilitate the transcription of NFκB target genes [34]. These include the gene encoding IkBα, which once synthesised, removes NFκB proteins from the nucleus back to the cytoplasm, and terminates its transcriptional activity [87].

### 3.2 Non-canonical NFκB signalling

**Fig. 4:**
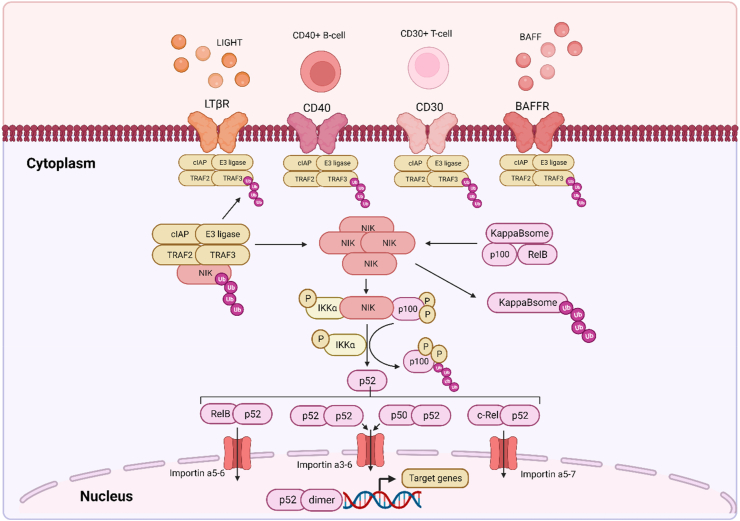
**The NFκB non-canonical signalling pathway:** This lesser activated pathway results in a slow and sustained signalling, culminating in systemic inflammation. Once activated by members of the tumour necrosis super family and immune system regulators, signalling proteins are recruited to the cellular membrane. This includes a complex consisting of; E3 ligase, cIAP, TRAF2 and TRAF3, which under unstimulated conditions resides in the cytoplasm, targeting NIK for ubiquitination. Once the pathway is stimulated, NIK accumulates and phosphorylates p100 and IKKα, where IKKα also phosphorylates p100, promoting its processing into p52. These phosphorylation's also result in the destruction of the kappaBsome-a complex which holds p100 and RelB in the cytoplasm. The now free p52 and RelB proteins form homodimers and heterodimers, which move into the nucleus via importin proteins, to bind to kappaB sites on the DNA to regulate the transcription of downstream target genes. **Abbreviations:** B-cell activating factor (BAFF), B-cell activating factor receptor (BAFFR), Tumour necrosis factor receptor superfamily member 8 (CD30), Tumour Necrosis Factor Receptor Superfamily Member 5 (CD40), cellular inhibitor of apoptosis protein 1 (cIAP), Tumour necrosis factor superfamily member 14 (LIGHT), lymphotoxin-β receptor (LTβR), NF-kappa-B-inducing kinase (NIK), Nuclear Factor Of Kappa Light Polypeptide Gene Enhancer In B-Cells 3 (RelB), TNF Receptor Associated Factor 2 (TRAF2), TNF Receptor Associated Factor 3 (TRAF3). Created in Biorender. com.

In contrast to the canonical pathway, the non-canonical pathway is more selective in its activation, producing a more specific response. These activators include members of the tumour necrosis receptor superfamily (TNFRSF), pattern-recognition receptors involved in innate immune system regulation, as well as adaptive immune system regulators; T and B cell receptors [62,88], as illustrated in Fig. 4. These stimuli result in a slow and persistent activation of the non-canonical signalling pathway. While the activation of the canonical pathway serves to induce the short-term transcription of pro-inflammatory target genes, activation of the non-canonical pathway produces a more sustained response, apt for its role in immune cell development and regulation [64].

In the absence of activation, the central kinase NFκB inducing kinase (NIK) is kept under tight regulation by E3 ubiquitin ligase dependent degradation. The E3 ligase forms a complex composed of cellular inhibitor of apoptosis (cIAP), TNFR-associated factor 2 (TRAF2) and TRAF3. Within the complex TRAF3 binds with NIK, and cIAP targets the kinase for ubiquitination and maintaining a low level of NIK in the cytoplasm [89].

Upon activation of the pathway via ligand receptor binding, the E3 ligase complex is quickly sequestered to the activated receptor [90], where cIAP targets the TRAF proteins for ubiquitination, rather than NIK. This change in cIAP's ubiquitination target is driven by TRAF2, allowing for NIK to accumulate in the cytoplasm [91].

Unlike other kinases in this pathway, NIK is constitutively active, and once able to accumulate in the cytoplasm, acts upon both p100 and IKKα. In contrast to the canonical pathway where there is evidence of functional redundancy [28,40,92], IKKα is the sole IKK protein to function in the non-canonical pathway. This phosphorylation promotes the recruitment of IKKα to p100, where both NIK and IKKα phosphorylate p100.

Prior to pathway activation, p100 is sequestered in the cytoplasm of the cell, with other NFκB proteins, such as RelB, in a complex commonly called the kappaBsome [93]. Once activated NIK binds to p100, at both serine 866 and 870, which then allows for the recruitment and subsequent phosphorylation of IKKα, at serine 176 by NIK [94]. Active IKKα phosphorylates p100 at serine 99, 108, 115, 123 and 872, resulting in the recruitment and binding of the SCF^βTrCP^ ubiquitin ligase, resulting in p52 production [95]. Christian et al. [72] reported that other p100 phosphorylation sites are active during p100 processing, however, the specific kinase responsible has not been identified.

The phosphorylation of NIK and IKKα results in the destruction of the kappaBsome and p100 processing cleaves the ankyrin repeats from p52, removing cytoplasmic retention and allowing for p52 to move into the nucleus [96]. Crystal structure studies have shown that p52 preferentially forms dimers with RelB or other p52 monomers and that the less frequent p52: c-Rel and p52: p50 heterodimers are less stable and more cell type specific [97].

The resulting dimers are subsequently imported into the nucleus through binding to importin proteins; p52 binding to importin a3-6, RelB to a5-6, and c-Rel binding to a5-7, via nuclear localisation signals [98]. Once inside the nucleus, these NFκB proteins bind to specific DNA consensus sequences known as kappaB sites, resulting in the transcription of pro-inflammatory and immune cell regulatory genes, as extensively outlined by Sun SC [99].

## 4 NFκB and associated proteins: expression in CRC and outcome

Both the canonical and non-canonical NFκB pathway drive the transcription of pro-inflammatory genes and are often described as the central regulator of inflammation. The aetiology and development of CRC has been intrinsically linked with inflammation [100,101], and research attention has turned to the role of NFκB. However, there is conflicting evidence regarding the role of NFκB in CRC, and how this affects patient outcome.

It has been argued that NFκB is constitutively active in 40–50 % of CRC cases [102,103] with this number closer to 60–80 % in cellular models [104,105]. However, these studies either only focus on canonical p65 activity, or reference NFκB as a single entity, often oversimplifying CRC as one disease, rather than stratifying it by tumour subtype or location (e.g. rectal cancer). Due to this, the full picture of constitutive action of the canonical or non-canonical pathway in CRC requires further clarification. Within this aberrant NFκB activity, members of the NFκB family contribute to multiple hallmarks of cancer, such as cell death evasion, tumour promoting inflammation, angiogenesis, metastasis [23], as well as maintaining a pro-cancerous TME through cytokine production and immune cell modulation [106].

This pro-tumorigenic activity has been reported to influence patient outcome in CRC. In a retrospective study of CRC, moderate expression of phosphorylated TAK1 was associated with poor cancer-free survival, and this effect was potentiated in patients with wild-type BRAF status. Additionally, this study concluded that high cytoplasmic IKKβ was significantly associated with decreased cancer-specific survival, suggesting that members of the canonical NFκB pathway are markers of poor prognosis in CRC patients [107]. This has been validated by research from other groups, reporting that p65 and p50 were predictive of decreased overall survival and increased chemotherapy resistance [108]. This has implications within the stratification of targeted therapies, as illustrated by a study investigating neoadjuvant therapy in CRC. They demonstrated patients expressing high p65 were less responsive to cetuximab and irinotecan than patients with low p65expression. This is thought to be due high levels of EGFR signalling activating NFkB, which is hypothesised to drive drug resistance by blocking apoptosis in these cells [109]. Furthermore, in a 51-patient cohort, high expression of the p65 gene associated with worse patient outcome and increased metastatic disease risk [110].

Another recent study illustrated that p65 is activated in response to POTE (Prostate, Ovary, Testis-Expressed Protein) and Sphingosine kinase 1 signalling. In a 20-patient study, it was demonstrated that high POTE gene expression was associated with poor patient survival, which was suggested to be orchestrated by p65 signalling [Shen et al., 2019]. This study illustrates that it may not be high expression levels of p65 protein that drives poorer patient outcome, but rather their activation by increased expression of upstream effectors, such as POTE.

In contrast there is evidence that IKKβ may act in a tumour suppressive manner, under certain conditions. A 2015 study found that within a model of colitis-associated cancer, fibroblast specific deletion of IKKβ caused increased epithelial proliferation, reduced cancer cell apoptosis and increased tumour growth [111]. This study contradicts most evidence within the field, however, does illustrate the importance of investigating NFκB's role and relationship with the TME.

Additionally, the non-canonical pathway has also been associated with poor patient prognosis. When associated with the cytokine LIF, IKKα was associated with poor CRC patient prognosis. This paper also reported *BRAF* dependent IKKα activity, which when inhibited, enhanced the chemosensitivity to a 5-FU based treatment in CRC cells [112]. In addition, Patel et al. also reported IKKα expression as a prognostic marker of poor patient survival, but interestingly, only when the kinase was observed to be clustered in distinct “punctate” areas, rather than consistently through the cytoplasm [113]. Taken together, the *BRAF* dependent mechanism of IKKα and punctate expression suggest alternative methods of IKKα signalling in CRC, which may lie outside of the non-canonical pathway. IKKα has been shown to have a key role in cancer development and progression within other solid cancers as clearly outlined by Colomer et al. [114]. Their review highlighted that IKKα has been associated with NFκB independent signalling in cancer, across KRAS mutant lung adenocarcinoma cells, basal cell carcinoma cells and breast cancer progenitors. The authors suggested that IKKα could aid pro-tumorigenic transformations, such as dampening p53 target gene transcription [115] and regulating genes associated with development and stemness [53,116]. Evidence such as this provides rationale for investigation into oncogenic and developmental pathways within CRC, to elucidate further mechanisms.

Conversely, there is evidence to support non-canonical NFκB signalling with anti-cancer effects in CRC. Data from a 217 CRC case study illustrated those activators of the non-canonical signalling pathway such as CD40, RANK, LIGHT and BAFF-R were downregulated in CRC tumours compared to adjacent normal tissue. This same study reports that NIK, the critical kinase for the non-canonical pathway was also downregulated, with the authors hypothesising that the non-canonical pathway is involved in beneficial inflammation, which is dampened in CRC [117]. Further studies have determined a protective role of IKKα in CRC, through conducting a meta-analysis examining NFκB gene expression. Their results illustrated that non-canonical signalling was decreased in CRC cells, which was further confirmed in patient CRC biopsies, when compared to adjacent normal tissue [118].

When discussing NFκB signalling it should be noted that there is far less data available regarding the non-canonical signalling pathway, compared to its canonical counterpart. However, the conflicting results from the non-canonical pathway cast doubt upon its role in CRC, and if this is a viable drug target or prognostic biomarker. As explored in further sections, there is a need to examine non-canonical NFκB data closely to gain the nuances of this pathway; what tissue types is this signalling present in? How does *KRAS* and *BRAF* status affect signalling and outcome? What is the role of NFκB within the TME, and how does this impact patient survival?

## 5 Targeting canonical and non-canonical signalling in CRC

Due to the evidence in support of the canonical pathway as an initiator and driver of CRC, drug discovery programs arose to target the central kinase of this signalling pathway IKKβ. X-ray crystallographic studies identified a promising binding site for inhibitors of IKKβ, which would result in a highly selective non-ATP competitive drug [119]. However, from the multitude of studies aiming to develop an inhibitor, no IKK inhibitor has passed a phase 2 clinical trial, as outlined by Ramadass et al. [120].

The reasons behind this have been extensively outlined by Prescott and Cook [121], discussing the lack of IKKβ selectivity within the drug, difficulties selecting appropriate patient groups that would benefit from such inhibitors, a lack of understanding of IKKβ′s contribution to the disease state, and safety concerns. Mouse studies have shown that IKKβ double knockout is embryonically lethal [122,123]. While IKKβ inhibition in humans is less severe, multiple IKKβ clinical trials have failed due to dose limiting toxicities such as thrombosis, esophagitis, and multiple reports of unfavourable safety profiles at phase 1. While many of these inhibitors are highly specific and potent, and essential tools in pre-clinical research, none have made it to the clinic [121].

Other drugs which target canonical signalling, outlined by Ramadass et al. have been investigated, however many of these have limited success in solid cancers, and are also associated with adverse side effects and safety issues [119]. Their review highlights that with further understanding of the pathway, more NFκB inhibiting drugs will pass through clinical trials, and this research effort has led to the positive effect of multiple drugs being repurposed in other clinical trials.

Within the non-canonical pathway, there have been pre-clinical attempts to drug NIK [124]. Due to the lack of success with IKKβ inhibitors, research interest has shifted to the other IKK, IKKα [125]. This includes the development application of plant flavone apigenin, as an IKKα and p65 inhibitor, which supresses tumour growth in prostate xenograft mice models [126]. Another strategy has looked at blocking IKKα′s translocation into the nucleus, such as using a glucosamine derivative, NCPA, which decreased markers of invasion and metastasis in osteosarcoma cell lines [127]. Furthermore, Anthony et al. have developed an IKKα specific inhibitor, which avoids the off-target effect of inhibiting IKKβ, in osteosarcoma and mouse embryonic fibroblasts [128,129], demonstrating promising results for an IKKα specific inhibitor.

While advances are being made in drugging NFκB in CRC, and other malignancies, issues such as a lack of specificity and off target effects hinder the process. This highlights a need to understand each component of the NFκB pathway, and their interactions with each other and the surrounding TME, to guide patient selection and drug development further.

## 6 IKKα outside of canonical and non-canonical signalling

From the attempts to target IKKβ and the canonical pathway, and the preliminary evidence from targeting IKKα, it is clear that an understanding of both the pathways, and any alternative signalling is required to overcome the problem of specificity. There has been growing interest in the field for investigating NFκB pathway members, out with canonical and non-canonical signalling. IKKα has been of particular interest, as many researchers now consider NIK the primary kinase of the NFκB pathway [124]. It has been suggested that IKKα may have a compensatory role or be involved in rarer pathways of its own [130], which will be explored in this section, focusing on pathways involved in CRC.

The interest in IKKα out of canonical and non-canonical signalling has been investigated by Cook et al. Their recent work illustrated that in HCT116 CRC cells, IKKα, rather than IKKβ, was the dominant kinase for facilitating NFκB activity in response to inflammatory cytokines TNFα and IL-1, not IKKβ. Within HCT116 and SW620 IKKα knockouts, IKKβ signalling was inefficient in compensating for the loss of IKKα, but in IKKβ knockouts, IKKα could compensate for the lost IKKβ activity [31]. This work highlights that although IKKα is not the dominant kinase for the canonical pathway, it was able to propagate the pathway within HCT116 cells, which carry a *KRAS* mutation. This indicates that there may be alternative pathways driven by IKKα in CRC, which exist outside of the canonical and non-canonical dogma.

Additionally, work in prostate cancer has illustrated that IKKα can drive tumour growth in TRAMP (transgenic adenocarcinoma of the mouse prostate) mice. When activated by RANKL (receptor activator of nuclear factor kappa beta ligand), IKKα can inhibit the expression of Maspin-a metastasis suppressor [131]. The study elegantly illustrated that if IKKα was inactivated (via a mutation), that tumour growth and metastasis decreased in the mice. However, this protection is abolished once Maspin was removed from the system [132]. This could be of particular interest in CRC, as RANKL is often produced by T cells and tumour associated macrophages in the immune rich environment which is present in right sided CRC, an aggressive subtype that requires novel intervention [133].

Furthermore, alternative forms of IKKα have been identified. Work by the Margalef and Colomer group has identified an alternative form of IKKα, which has interesting implications in CRC. In 2012 the group analysed 288 CRC patient samples and found that most tumours exhibited high levels of phosphorylated IKKα (serine 180). There was a high level of cytoplasmic immunohistochemical staining in discrete cytoplasmic vesicles-which from further work was shown to be a truncated version of IKKα, termed p45-IKK due to its molecular weight. This truncated form was associated with increased tumour grade, and *in vitro* and *in vivo* work illustrated that the processing of IKKα into p45-IKK was essential for tumour growth and apoptosis evasion. Furthermore p45-IKK was associated with markers of endosomal compartments, suggesting possible processing by the Golgi apparatus [134].

This pattern of staining is consistent with the observations from other groups, such as a study of 1030 stage 2–3 CRC patients by Edwards et al. In this patient cohort discrete cytoplasmic staining (termed punctate in this paper) was associated with poor cancer-specific survival, and *KRAS* mutation. Upon investigation by immunofluorescent staining, it was demonstrated that the punctate IKKα staining co-localised with the Golgi apparatus [113]. It is hypothesised that punctate staining is indicative of p45-IKK and has implications in advanced CRC.

Other studies have identified associations with IKKα and endosomal compartments, such as a 2018 study which found IKKα recruitment to the endosomes was essential for the production of cytokine type 1 interferon, hypothesised to promote inflammation and immune cell regulation [135]. It is possible that this signalling is present in p45-IKK containing CRC cells, driving systemic inflammation and a pro-tumourgenic TME.

Margalef and Colomer et al. expanded their p45-IKK work in 2015, illustrating that mutant *BRAF*, but not mutant *KRAS*, triggers the activation of p45-IKK through TAK1 signalling, rather than activating the canonical NFκB pathway [136]. Interestingly, they found that TAK1 phosphorylated p45-IKK and was associated with endosomal compartments. Blocking this phosphorylation, by disrupting endosomal V-ATPase, reduced growth, and metastasis of mutant *BRAF* xenografts in mice [137], suggesting that this previously unknown pathway could be targeted in CRC, specifically targeting p45-IKK or their associated endosomes.

It should be noted that the Edwards group reported cytoplasmic punctate staining was associated with *KRAS* mutations, and Margalef and Colomer found that *BRAF* mutations were associated with a high level of nuclear phosphorylated IKKα. This may be due to differences between cytoplasmic and nuclear IKKα\ IKKα isoform expressions, or that mutant *KRAS* induces wildtype *BRAF* signalling in CRC, resulting in p45-IKK signalling. Further investigation of this isoform across mutational subtypes is required.

Leading on from this *BRAF*-TAK1-p45-IKK work, Margalef and Colomer found that in response to chemotherapy and radiotherapy DNA damage, p45-IKK is activated by *BRAF*-TAK1 and p38-MAPK, allowing for ataxia-telangiectasis mutated (ATM) kinase driven DNA damage response [138]. They hypothesised that this could be a mechanism for IKKα driven therapy resistance [139] and found that inhibition of IKKα (theorised to also inhibit p45-IKK) or *BRAF* significantly increased the effects of 5-FU and irinotecan in patient derived CRC tumoroids.

The evidence of a truncated form of IKKα that is present in CRC, and may be *KRAS* or *BRAF* dependent, may explain variations in reports regarding IKKα′s prognostic value, where reported total IKKα may be a combination of IKKα and p45-IKK. Alternative pathways may be valuable drug targets or prognostic markers for patients with advanced CRC, where p44-IKK should be treated independently from IKKα.

## 7 Future perspectives

The field of NFκB signalling has evolved dramatically over the past couple of decades, and is reaching a translational tipping point in CRC [140]. Evidence of alternative signalling pathways, which may be influenced by mutational status, present an exciting avenue for therapeutic intervention. However, when looking at the history of targeting the IKKs for therapeutic intervention, it is clear that there is a need delineate these alternative signalling pathways, to avoid issues of drug side effects and non-specificity.

The use of novel methods, such as spatial transcriptomics could help examine these underlying pathways. In a study looking another key inflammatory pathway, JAK/STAT3, Nanostring GeoMx® digital spatial profiling was utilised to investigate the different spatial compartments of high STAT3 expressing tumour microenvironment in triple negative breast cancer tumours. The study found a subpopulation of high STAT3 tumours which had a unique gene expression profile, and a stromal rich-immune- deficient phenotype [141], which was associated with poor prognosis. This could be applied to IKKα high CRC patient groups, in order to elucidate if there are drug targetable subgroups present, and gain mechanistic information to aid drug development.

Furthermore, the association between NFκB and non-coding RNAs, such as microRNA (miRNA) or long non-coding RNA (lncRNA) within CRC should also be considered. As NFκB is a family of transcription factors, and miRNAs offer a route of dysregulation through transcriptional regulation, there has been significant research performed to investigate these interactions [142]. The wealth of information regarding CRC, NFκB and their interactions with non-coding RNA is beyond the scope of this article, however, does offer some interesting future perspective. One such avenue of investigation has studied the use of non-coding RNAs as liquid biopsies; a 2021 study in breast cancer illustrated that specific non-coding RNA (LINC00511), and its downstream miRNA (miR-185–3 P) were correlated with lymph node metastasis and advanced tumour grade [143]. This has also been investigated in CRC, as extensively outlined by Yang et al. highlighting potential biomarkers such as long non-coding RNA FGD5-Antisense RNA 1, levels of which are significantly increased in 5-FU resistance cells [144]. Interestingly miRNA have also been associated with multi-drug resistance, with different miRNA expression patterns shown between drug resistant and susceptible cells [145]. This should be taken into consideration when examining patient response to any resulting IKKα inhibitors, as a miRNA signature of response (or resistance) would aid the successful stratification of patients.

## 8 Strengths and limitations of the article

This article aimed to highlight the advances in the recent decade within investigating NFκB signalling and discuss this within the scope of CRC and its treatment. As CRC poses a significant strain on the global population, there is a need to evaluate the field of NFκB signalling in CRC and use this wealth of evidence to guide the development of novel therapeutics, which this review has discussed.

However, we are aware of limitations to this article. Firstly, this review focuses on only one cancer type, CRC. As discussed throughout the article, NFκB signalling has been shown to vary across cell type and importantly tissue type, emphasising that to apricate the full scope of NFκB signalling, multiple cancer types should be considered [146]. Secondly this review does not discuss the complexities and detailed research occurring within the field of non-coding RNAs and NFκB. There is a wealth of evidence highlighting interactions between the two, which unravel mechanistic, prognosis and therapeutic questions, as outlined by Mirzaei et al. Mahmoud et al., and Emam et al., [147]; Mahmoud et al., 2021a [148]. As previously mentioned, this is not within the scope of this review. Additionally, crosstalk between key pathways, such as JAK/STAT, have not been covered in this review, which have been highlighted within the literature as a key consideration when designing therapeutic interventions [149]. Finally, we appreciate that cancer is not the only condition where inflammation and NFκB are integral. Further discussion of NFκB in other inflammatory diseases, such as diabetes, multiple sclerosis and rheumatoid arthritis as outlined by Liu et al. [62] is required.

## 9 Conclusions

There have been numerous advances in NFκB research within CRC, with the understanding of the multiple signalling components and pathway crosstalk increasing, and more prognostic data emerging for individual pathway components. This has been investigated within inflammation, due it its long-established links with CRC, as a known driver of tumour initiation, development and drug resistance. Prognostic data examining NFκB and IKK expression in CRC has shown conflicting results, possibly due to variations in NFκB across cell and tissue type, and the diverse functions of NFκB in CRC. Therefore, there is a need to examine specific NFκB signalling components across different cell types, to understand what signalling is occurring in which subsets of CRC patients.

Although it has been well established that IKKβ is the driving kinase of the canonical pathway, as more non-canonical data has become available, it has been suggested that NIK is the driving kinase for the non-canonical pathway, rather than IKKα. This has implications for drug development, as due to the lack of efficacy and safety issues faced by IKKβ inhibitors, efforts have turned to inhibiting IKKα. While this kinase does have a role in non-canonical signalling, it has been demonstrated that it has a central role in canonical signalling and is involved in other intracellular signalling cascades in CRC, such as the *BRAF*-TAK1-p45IKK-IKKα signalling axis. Further investigation into pathways outside of canonical and non-canonical signalling may provide insight into how NFκB family members signal within CRC, and provide novel targets, or critical information, for drugging these pathways of inflammation in CRC.

## CRediT authorship contribution statement

**Molly McKenzie:** Writing – review & editing, Writing – original draft, Visualization, Software, Resources, Project administration, Investigation, Conceptualization. **Guang-Yu Lian:** Writing – review & editing. **Kathryn A.F. Pennel:** Writing – review & editing. **Jean A. Quinn:** Writing – review & editing. **Nigel B. Jamieson:** Writing – review & editing, Supervision. **Joanne Edwards:** Writing – review & editing, Supervision, Conceptualization.

## Declaration of competing interest

The authors declare that they have no known competing financial interests or personal relationships that could have appeared to influence the work reported in this paper.
